# The complete chloroplast genome of *Eleutherococcus trifoliatus* (Araliaceae): a wild edible plant in the coastal region of South China

**DOI:** 10.1080/23802359.2019.1707131

**Published:** 2020-01-14

**Authors:** Sheng Chen, Yuan Xu, Dan Liang, Ruijiang Wang

**Affiliations:** Key Laboratory of Plant Resources Conservation and Sustainable Utilization, South China Botanical Garden, Chinese Academy of Sciences, Guangzhou, China

**Keywords:** *Eleutherococcus trifoliatus*, chloroplast genome, wild vegetable, phylogenetic analysis

## Abstract

*Eleutherococcus trifoliatus* (L.) S. Y. Hu is a wild edible plant and widely used in the coastal region of South China. Here, we report the complete chloroplast genome of *E. trifoliatus*. The length of the cp genome was determined to be 156751 bp with a small single copy (SSC) region of 18316 bp, a large single copy (LSC) region of 86747 bp and two separated inverted region of 25844 bp, respectively. Totally, 132 unique genes were identified of this genome, including 87 protein-coding genes, 37 tRNA genes and eight rRNA genes. The GC contents of this genome is 38%. Chloroplast phylogenomics analysis indicates that *E. trifoliatus* is closely related to *E. gracilistylus* (W.W. Sm.) S.Y. Hu.

*Eleutherococcus trifoliatus* (L.) S. Y. Hu is a commonly used wild vegetable and tea in the coastal region of South China (Wang et al. 2015; Li et al. 2017). It belongs to the ginseng family (Araliaceae) and distributed in South China, India, Japan, Philippines, Thailand and Vietnam (Xiang and Lowry 2007). *Eleutherococcus* is one of the important medicinal genera of Araliaceae (Wen et al. 2001), but the phylogenetic relationship between *Eleutherococcus* and its related genera still uncertain (Li and Wen 2016). Thus we sequenced the complete chloroplast (cp) genome of *E. trifoliatus* to elucidate its phylogenetic relationship in the family.

The total genomic DNA was extracted from silica gel-dried leaf sample following the CTAB method (Doyle and Doyle 1987). Leaf sample of *E. trifoliatus* was collected from Nan Ao Island in Shantou City, Guangdong Province, China (117°08′10″E, 23°26′29″N). The voucher specimens (accession number: 0849959) was deposited in the herbarium of South China Botanical Garden (IBSC). The genomic library (paired-end, PE = 150 bp) was sequenced on a BGISEQ-500 platform at Beijing Genomics Institute (Shenzhen, China). Totally, 2 Gb sequence reads were obtained and used to assemble the cp genome after filtering and trimming the low quality reads and adaptor sequences. The annotated plastome for *Metapanax delavayi* was downloaded from GenBank (accession number: NC_022812), and was used as the reference for the complete cp genome assembly and annotation. Reference assembly was executed on NOVOPlasty 2.6.3 (Dierckxsens et al. 2017) with kmer length set to 39 base pairs. Annotation of the cp genome was performed on Geneious version 11.0.3 (Kearse et al. 2012), while the tRNA genes were annotated on ARAGORN (Laslett and Canback 2004).

The complete cp genome of *E. trifoliatus* (GenBank accession number: MN727298) was 156751 bp in length, which composed of a small single copy region (SSC) of 18316 bp, a large single copy region (LSC) of 86747 bp and a pair of inverted repeats (IRs) of 25844 bp. The genome encodes a total of 132 unique genes, including 87 protein-coding genes, 37 tRNA genes and eight rRNA genes. Among these genes, 17 genes has two copies, which including six protein-coding genes, seven tRNA genes and four rRNA genes. The GC contents of this genome is 38%, while the GC contents of LSC, SSC and IRs are 36.2%, 32.1% and 43.1% respectively.

To clarify the phylogenetic position of *E. trifoliatus*, we reconstructed the phylogeny of *Eleutherococcus*-*Dendropanax*-*Schefflera* Group of Araliaceae (Wen et al. 2001) based on all of the reported plastomes of this group. The plastomes of the 14 accessions were aligned using MAFFT (Katoh and Standley 2013). Then, the maximum likelihood (ML) tree was reconstructed using RAxML (Stamatakis 2006) with 1000 bootstrap replicates. Based on the topology of the phylogenetic tree (Figure 1), all of the studied *Eleutherococcus* species form a monophyletic clade. *Eleutherococcus trifoliatus* is closely related to *E. gracilistylus* (W.W. Sm.) S.Y. Hu.

**Figure 1.**
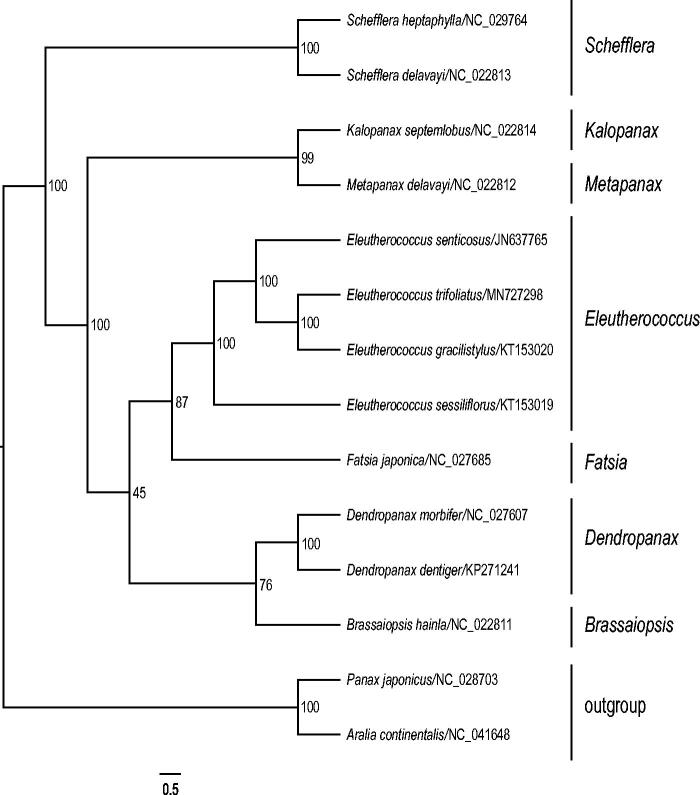
Maximum likelihood tree based on 14 complete chloroplast genomes of Araliaceae. Bootstrap support values are shown at the branches.

## References

[CIT0001] Dierckxsens N, Mardulyn P, Smits G. 2017. NOVOPlasty: De novo assembly of organelle genomes from whole genome data. Nucleic Acids Res. 45(4):e18 28204566 10.1093/nar/gkw955 PMC5389512

[CIT0002] Doyle JJ, Doyle JL. 1987. A rapid DNA isolation procedure for small quantities of fresh leaf tissue. Phytochem Bull. 19:11–15.

[CIT0003] Katoh K, Standley DM. 2013. MAFFT multiple sequence alignment software version 7: improvements in performance and usability. Mol Biol Evol. 30(4):772–780. 23329690 10.1093/molbev/mst010 PMC3603318

[CIT0004] Kearse M, Moir R, Wilson A, Stones-Havas S, Cheung M, Sturrock S, Buxton S, Cooper A, Markowitz S, Duran C, et al. 2012. Geneious basic: an integrated and extendable desktop software platform for the organization and analysis of sequence data. Bioinformatics. 28(12):1647–1649. 22543367 10.1093/bioinformatics/bts199 PMC3371832

[CIT0005] Laslett D, Canback B. 2004. ARAGORN, a program to detect tRNA genes and tmRNA genes in nucleotide sequences. Nucleic Acids Res. 32(1):11–16. 14704338 10.1093/nar/gkh152 PMC373265

[CIT0006] Li DL, Zheng XL, Duan L, Deng SW, Ye W, Wang AH, Xing FW. 2017. Ethnobotanical survey of herbal tea plants from the traditional markets in Chaoshan, China. J Ethnopharmacol. 205:195–206. 28249822 10.1016/j.jep.2017.02.040

[CIT0007] Li R, Wen J. 2016. Phylogeny and diversification of Chinese Araliaceae based on nuclear and plastid DNA sequence data. J Syst Evol. 54(4):453–467.

[CIT0008] Stamatakis A. 2006. RAxML-VI-HPC: maximum likelihood-based phylogenetic analyses with thousands of taxa and mixed models. Bioinformatics. 22(21):2688–2690. 16928733 10.1093/bioinformatics/btl446

[CIT0009] Wang H, Li D, Du Z, Huang MT, Cui X, Lu Y, Li C, Woo SL, Conney AH, Zheng X, et al. 2015. Antioxidant and anti-inflammatory properties of Chinese ilicifolius vegetable (*Acanthopanax trifoliatus* (L) Merr) and its reference compounds. Food Sci Biotechnol. 24(3):1131–1138.

[CIT0010] Wen J, Plunkett GM, Mitchell AD, Wagstaff SJ. 2001. The evolution of Araliaceae: a phylogenetic analysis based on ITS sequences of nuclear ribosomal DNA. Syst Bot. 26(1):144–168.

[CIT0011] Xiang QB, Lowry PL. 2007. Araliaceae. In: Wu ZY, Raven PH, Hong DY, editors. Flora of China. vol. 13. St. Louis (MO): Science Press, Beijing and Missouri Botanic Garden Press; p. 435–491.

